# High Toned Quackery

**Published:** 1872-07

**Authors:** Wm. R. McLean


### HIGH TONED QUACKERY.

WM. R. M'LEAN, M. D.

If, by the term quackery, we mean imposition and deception, I do not think we would be uncharitable in saying that quackery is universal. We cannot always tell how much of a quack a man is by the length of his hair; but we do think we can tell it a physician has been treating a patient for a condition of things that did not exist. It is the duty of the physician to be honest with his patients, and if, through ignorance or carelessness, he goes on week after week in the same channel, what else can we call it but a form of quackery. I have often been told where I had visited different members of the family, at different times, by the mother, "Doctor, I am discouraged, *utterly so!* if I had the least confidence in your helping me, I would place myself under your care, but I have doctored with Dr. —— and you know everybody knows of him; he owns a marble front in the city, and has *so much* practice!" From this she argues that the physician who lives in an ordinary pine front, and having a moderate practice is not supposed to know anything.

I trust that we have the light of modern days and a consciousness of what constitutes the true physician before our eyes.

I have had unusual opportunities for discovering that quackery is found among doctors of high tone and eminent in the profession, as well as among those of less pretentions and moderate incomes.

I will state a case, and the reader may form his own opinion: A most estimable lady had been subject to very profuse menstruations, almost lasting from one period to another, for which she was treated by a celebrated doctor for ulceration of the uterus; the nitrate of silver being applied weekly. Growing no better under the treatment, her friends urged her to change physicians. Her physician being a man of years, and residing in the afore-mentioned marble front, of course, was considered infallible, and was retained. In the mean time the patient kept growing worse, until a violent hemorrhage occurred. The friends not having time to summon her physician, a friend volunteered to send for me. I soon made my appearance, and having learned the condition of things, took my own way in managing the case, avoiding anything like excitement or alarm.—Often, when the patient could summon strength to speak, she would say, "her physician did not do thus and so." I would have been discharged and recalled a dozen times if her husband had not insisted on my carrying the case through. With some difficulty I checked the hemorrhage, and left my patient comfortable, stating to the husband that I feared the existence of a polypus, and requested the privilege of returning the next morning to make the proper examination, which was granted.—On making the examination, I found a granular polypus slightly protruding through the cervix to which the illustrious physician had been applying his caustic for ulceration. Having taken time to dilate the cervical canal, I severed the pedicle of the polypus with my scissors, putting an end to her hemorrhage.

I have not the vanity to claim any peculiar talent, skill, or any other merit for the favorable termination of the above case, save that that would have been used by any modest practitioner of sense or judgment, notwithstanding his lodgment in a plain cottage of unassuming proportions.

The young physician need not be discouraged, as it requires more than elaborately formed technical words to secure patrons. He must keep posted, and above all, be strictly conscientious in all his dealings with suffering humanity, if he would be an honorable and successful practitioner of medicine.

